# Reduced risk of severe COVID-19 with lithium use: a large-scale comparison with valproate users and other COVID-19 patients

**DOI:** 10.3389/fpsyt.2025.1702189

**Published:** 2026-01-05

**Authors:** Chen Avni, Uri Blasbalg, Paz Toren

**Affiliations:** 1 Ramat-Chen Brüll Mental Health Center, Tel-Aviv District, Clalit Health Services Community Division, Tel Aviv, Israel; 2 Gray Faculty of Medical & Health Sciences, Tel Aviv University, Tel Aviv, Israel

**Keywords:** COVID-19, clinical outcomes, lithium, valproate, ECMO, vaccination

## Abstract

**Background:**

Lithium, a commonly used mood stabilizer with immunomodulatory and antiviral properties, has been hypothesized to lessen the severity of COVID-19, but population-level evidence remains limited.

**Methods:**

In this retrospective cohort study, we examined electronic health records from over 1.49 million adults with confirmed COVID-19 in Israel between March 2020 and November 2021. Individuals were categorized based on sustained use of lithium (n=857), valproate (n=5,302), or no exposure to either medication. Severe COVID-19 was defined as hospitalization, respiratory support, extracorporeal membrane oxygenation, or death. To account for baseline differences, we applied inverse probability weighting and logistic regression with correction for rare events.

**Results:**

Lithium use was associated with significantly lower odds of severe COVID-19, even though users were older and had more medical comorbidities. In contrast, valproate use was linked to increased risk.

**Conclusion:**

These findings suggest that lithium may provide a protective effect against severe COVID-19 outcomes, independent of underlying vulnerability, and support further investigation into the broader health implications of psychotropic medications.

## 1 Introduction

People with severe mental illness (SMI) face disproportionately poor COVID-19 outcomes, with schizophrenia spectrum disorders in particular being associated with markedly elevated mortality risk independent of cardiometabolic comorbidities (1–3). This vulnerability highlights the importance of evaluating how psychotropic treatments interact with SARS-CoV-2 pathophysiology in this population.

The COVID-19 pandemic, which emerged in late 2019, posed unprecedented challenges to global healthcare systems and spurred the scientific community to seek effective therapeutic solutions. Among these efforts, existing compounds with potential antiviral properties have garnered research attention, including lithium. Lithium is a long-standing medication primarily used to treat mood disorders such as bipolar disorder. Since the 1980s, numerous studies have suggested that lithium has antiviral efficacy. For instance, early research indicated lithium’s ability to inhibit the replication of certain viruses, such as herpes simplex virus (4). More relevantly, a large-scale study of real-world data, prior to the pandemic, demonstrated that lithium treatment was associated with a reduced risk of respiratory infections, whereas valproate exposure was linked to an increased risk (5). Lithium has also been noted for its capacity to modulate immune responses and reduce inflammation resulting from viral infections (6). Beyond these broad immunomodulatory actions, lithium directly inhibits glycogen synthase kinase-3 (GSK-3). Phosphorylation of the coronavirus nucleocapsid (N) protein by GSK-3 is required for efficient viral replication and assembly, providing a specific mechanistic rationale for lithium’s potential antiviral activity against SARS-CoV-2 (7). A systematic review and meta-analysis of psychotropic agents in the context of COVID-19 highlighted lithium as a candidate for repurposing, noting both mechanistic plausibility and preliminary clinical support for protective effects (8). Together, these findings support lithium’s relevance in infectious disease but leave unanswered whether such associations extend to COVID-19 severity in modern clinical settings, including vaccinated populations.

With the advent of the COVID-19 pandemic, several studies explored the potential application of lithium in combating SARS-CoV-2 (9, 10). Evidence regarding lithium and COVID-19 has been mixed. While a small randomized trial suggested lithium reduced hospitalization and mortality (11), several small studies raised concerns about increased mortality rates, toxicity, and destabilization during infection (12–14). A large retrospective study of over 26,000 lithium users suggested a lower incidence of infection compared to 100,000 valproate users, hinting at a potential protective effect (15). More recently, a nationwide Swedish register study of over 39,000 individuals with bipolar disorder found no significant association between lithium treatment and COVID-19–related mortality, hospitalization, or ICU admission after adjustment for confounders. Notably, in an exploratory subgroup analysis, lithium monotherapy was associated with reduced risks of hospitalization and ICU admission compared to combination therapy, suggesting that protective effects may depend on specific treatment contexts or patient subgroups (16).

Because valproate has also been hypothesized, though inconsistently demonstrated, to influence immune and inflammatory pathways through histone deacetylase (HDAC) inhibition and related mechanisms, it represents a meaningful active comparator. Examining lithium against valproate, alongside individuals unexposed to either drug, may help clarify whether observed associations reflect mood-stabilizer use in general or lithium-specific biological effects (17).

In light of the conflicting findings and the emerging therapeutic potential, there is a need for more in-depth and comprehensive research to better understand the relationship between lithium treatment and the consequences of the COVID-19 pandemic. Such research could clarify whether lithium’s potential benefits extend to the severity of COVID-19 outcomes, particularly in the context of vaccinated populations and modern treatment settings, while also helping to identify subgroups most likely to benefit and evaluating potential risks.

## 2 Materials and methods

The study was approved by the institutional review boards (Study designation 0070-23-COM1). It was conducted in accordance with the International Conference on Harmonization guidelines and ethical principles of the Declaration of Helsinki.

This retrospective cohort study was based on data from Clalit Health Services (CHS), the largest integrated healthcare provider in Israel, covering over 4.5 million members (approximately 54% of the Israeli population). The CHS electronic health record (EHR) database includes detailed longitudinal information on diagnoses from both hospital and outpatient settings, demographic attributes, medication purchase records, laboratory test results, and clinical procedures. The data were extracted in June 2024 via CHS’ secure platform, powered by MDClone, which allows access to anonymized datasets for research purposes. Extracted anonymized data were stored in a secure project library, accessible only to authorized researchers within the CHS research division.

### 2.1 Sample

The study population included adult CHS members aged 18 and older who tested positive for COVID-19, observed between March 1, 2020, and November 30, 2021. All individuals with available data on age, sex, socioeconomic status (SES), and COVID-19 vaccination status were eligible.

Exposure classification was based on medication purchase patterns. Individuals were categorized into three mutually exclusive groups:

1. Lithium group – those who purchased lithium in at least five of six quarterly periods during the study timeframe, indicating continuous regular use.

2. Valproate group – those who met an equivalent usage pattern for valproate.

3. Unexposed group (non-users) – those not exposed to either medication during the study window.

### 2.2 Variables

#### 2.2.1 Demographic characteristics

Age (continuous), gender (male/female), and SES were extracted for all individuals. SES was determined based on the classification of the patient’s primary care clinic using data from the Israeli Central Bureau of Statistics, categorized into low, medium, and high SES.

#### 2.2.2 Clinical and psychiatric comorbidities

Preexisting medical conditions were identified using ICD-10 diagnostic codes documented during or prior to the study period. The following medical conditions and history were included: obesity, schizophrenia/schizoaffective disorder, liver disease, cardiovascular disease, malignancy, COVID-19 vaccination status, antiviral therapy, hypertension, chronic obstructive pulmonary disease (COPD), diabetes, renal disease, immunodeficiency, and dementia. Each condition was coded as a binary variable indicating presence or absence.^1^.

#### 2.2.3 COVID-19 severity outcomes

The primary outcome was a composite indicator of severe COVID-19, defined as the presence of any of the following clinical events: hospitalization due to COVID-19, requirement for respiratory support, use of extracorporeal membrane oxygenation (ECMO), or death attributed to COVID-19. Each of these components was also analyzed independently as secondary outcomes. All outcomes were identified using COVID-19-specific ICD-10 diagnostic codes, procedure codes, and official death registry data.

### 2.3 Statistical analysis

Baseline characteristics were compared across exposure groups using standardized mean differences (SMDs), rather than hypothesis testing. This approach quantifies the magnitude of imbalance independent of sample size, with values <0.1 generally considered negligible, 0.1–0.2 small, 0.2–0.5 moderate, and >0.5 large. Given the very large reference group (>1.4 million individuals), formal statistical tests would render even trivial differences statistically significant. SMDs, therefore, provide a more appropriate and interpretable measure of baseline comparability.

Due to an imbalance in group sizes and the rarity of severe COVID-19 outcomes in the lithium group, a two-step approach was used:

1. Inverse Probability Weighting (IPW) was applied to estimate the Average Treatment Effect on the Treated (ATT). The propensity score model included 20 baseline covariates to balance potential confounders across the exposure groups. Weights were stabilized and trimmed to ensure robustness and avoid extreme values. Alternative techniques (ATE, entropy balancing) were tested but yielded excessive variability or imbalance.

2. Following IPW, a Firth-penalized logistic regression model was used to estimate the association between exposure status and the primary outcome, correcting for quasi-separation that arose due to the very low number of severe outcomes in the lithium group.

Secondary models estimated associations between exposure status and each severity component (hospitalization, respiratory support, ECMO, death) using the same IPW-weighted framework.

Results are reported as P-values, followed by odds ratios (ORs) with corresponding 95% confidence intervals (CIs).

## 3 Results

The final cohort consisted of 1,490,638 individuals who met the eligibility criteria and had complete data for all covariates. Among these, 857 had been prescribed lithium, 5,302 had been prescribed valproate, and the remainder (over 1.48 million) had no exposure to either agent during the assessment period.

**Table 1** presents the baseline characteristics across the three groups. The mean age was higher in the exposed groups compared to the unexposed, with individuals in the lithium group averaging 56.4 years and those in the valproate group averaging 53.9 years, versus 49.8 years in the unexposed population. The gender distribution also differed across groups, with a higher proportion of males in the valproate group (58%) compared to the lithium group (45%) and the unexposed group (43%).

**Table 1:** Characteristics of the sample by exposure group.

Characteristic	Overall (N = 1,490,638)	None (N = 1,484,479)	Valproate (N = 5,302)	SMD	Lithium (N = 857)	SMD
Demographics						
Age, mean (SD)	49.8 (18.3)	49.8 (18.3)	53.9 (17.4)	**0.22**	56.4 (16.3)	**0.36**
Male, n (%)	648,794 (44)	645,307 (43)	3,099 (58)	**0.3**	388 (45)	0.04
SES: Low, n (%)	234,329 (16)	233,369 (16)	873 (16)	0.02	87 (10)	–0.15
SES: Medium, n (%)	863,348 (58)	859,698 (58)	3,128 (59)	0.02	522 (61)	0.06
SES: High, n (%)	300,827 (20)	299,783 (20)	846 (16)	–0.11	198 (23)	0.07
SES: No data, n (%)	92,134 (6.2)	91,629 (6.2)	455 (8.6)	0.1	50 (5.8)	–0.01
Medical history						
Bipolar disorder, n (%)	10,801 (0.72)	9,376 (0.63)	812 (15.3)	**0.54**	613 (71.5)	**1.48**
Obesity, n (%)	101,643 (6.8)	101,069 (6.8)	467 (8.8)	0.08	107 (12)	**0.23**
Schizophrenia or Schizoaffective disorder, n (%)	18,795 (1.3)	16,815 (1.1)	1,622 (31)	**2.66**	358 (42)	**3.8**
Liver disease, n (%)	1,382 (<0.1)	1,374 (<0.1)	6 (0.1)	0.01	2 (0.2)	0.05
CVD, n (%)	131,259 (8.8)	130,539 (8.8)	635 (12)	0.11	85 (9.9)	0.04
Malignancy, n (%)	153,623 (10)	152,833 (10)	672 (13)	0.08	118 (14)	0.11
COVID-19 vaccination, n (%)	1,113,842 (75)	1,108,499 (75)	4,579 (86)	**0.27**	764 (89)	**0.33**
Antiviral therapy, n (%)	38,029 (2.6)	37,956 (2.6)	52 (1.0)	–0.10	21 (2.5)	–0.01
Hypertension, n (%)	118,782 (8.0)	118,209 (8.0)	487 (9.2)	0.05	86 (10)	0.08
Chronic Obstructive Pulmonary Disease (COPD), n (%)	64,459 (4.3)	63,962 (4.3)	440 (8.3)	**0.2**	57 (6.7)	0.12
Diabetes, n (%)	217,270 (15)	215,862 (15)	1,202 (23)	**0.23**	206 (24)	**0.27**
Renal disease	75,838 (5.1)	75,264 (5.1)	480 (9.1)	0.18	94 (11)	**0.27**
Immunodeficiency, n (%)	1,924 (0.1)	1,912 (0.1)	10 (0.2)	0.02	2 (0.2)	0.03
Dementia, n (%)	57,303 (3.8)	56,603 (3.8)	629 (12)	**0.42**	71 (8.3)	**0.23**

SMD, standardized mean difference versus the unexposed (None) group. Conventionally, SMD <0.1 = negligible, 0.1–0.2 = small, 0.2–0.5 = moderate, >0.5 = large imbalance. Moderate or larger SMD values were marked in bold for the reader’s convenience.

Socioeconomic status, obesity, psychiatric history, and physical comorbidities, including cardiovascular, hepatic, oncologic, and renal conditions, also varied across groups. Notably, individuals exposed to either psychiatric medication showed a higher prevalence of multiple chronic conditions.

Both lithium and valproate groups were older and more comorbid than the unexposed population, with SMDs indicating moderate to large imbalances for age, obesity, diabetes, renal disease, and psychiatric morbidity. Lithium patients in particular showed greater imbalance than valproate in several domains (e.g., obesity, schizophrenia, diabetes, renal disease), whereas valproate patients had higher COPD and dementia burden.

### 3.1 Association between severe COVID-19 and drugs exposure group

A weighted logistic regression model was fitted to estimate the association between psychiatric medication exposure and severe COVID-19 outcomes. Inverse probability weighting was used to account for baseline differences across the groups, trimming extreme weights to improve stability.

In this model, exposure to lithium was associated with a substantially lower risk of developing severe COVID-19. The adjusted odds ratio (OR) was 0.36 (95% confidence interval [CI]: 0.16–0.82, *p* = 0.015), indicating a 64% relative reduction in odds compared to unexposed individuals, after adjusting for age, sex, comorbidities, and socioeconomic status.

In contrast, exposure to valproate was associated with a more than twofold increase in the risk of severe COVID-19, with an OR of 2.40 (95% CI: 2.05–2.81, *p* < 0.0001).

As expected, older age, male sex, obesity, and various chronic conditions were independently associated with greater odds of severe outcomes while vaccination conferred lower risks of severe outcomes. The magnitude and precision of these associations are provided in **Table 2**.

**Table 2:** IPW-weighted logistic regression model predicting severe COVID-19 outcomes by exposure group and covariates.

Predictor	Estimate	Std. Error	*t* value	*p*-value	*OR*	95% Confidence Interval	
(Intercept)	-6.1263794	0.0377544	-162.269	< 0.001 ***			
Exposure							
Lithium	-1.0230183	0.4229315	-2.419	0.015 *	0.360	0.157	0.824
Valproate	0.8746562	0.0809403	10.806	< 0.001 ***	2.398	2.046	2.810
Demographics							
Age	0.042	0.001	73.258	< 0.001 ***	1.043	1.042	1.044
Gender (Male)	0.099	0.015	6.572	< 0.001 ***	1.104	1.072	1.137
SES – Medium	-0.428	0.017	-22.957	< 0.001 ***	0.652	0.629	0.676
SES – High	-0.507	0.025	-19.924	< 0.001 ***	0.602	0.573	0.633
SES – No data	-0.124	0.033	-3.744	< 0.001 ***	0.884	0.828	0.943
Medical history							
Obesity	0.324	0.022	14.393	< 0.001 ***	1.382	1.322	1.444
Schizophrenia or Schizoaffective disorder	0.706	0.043	16.460	< 0.001 ***	2.026	1.862	2.203
Liver disease	0.847	0.107	7.939	< 0.001 ***	2.333	1.893	2.876
Cardiovascular	0.514	0.019	27.407	< 0.001 ***	1.672	1.612	1.735
Malignancy	0.345	0.018	19.307	< 0.001 ***	1.412	1.363	1.462
COVID-19 Vaccination	-1.527	0.016	-98.019	< 0.001 ***	0.217	0.211	0.224
Anti-viral treatment	-0.696	0.046	-15.181	< 0.001 ***	0.499	0.456	0.545
Hypertension	0.603	0.018	34.192	< 0.001 ***	1.827	1.765	1.892
Chronic Obstructive Pulmonary Disease (COPD)	0.394	0.021	18.364	< 0.001 ***	1.482	1.421	1.546
Diabetes	0.333	0.017	19.663	< 0.001 ***	1.396	1.350	1.443
Renal disease	0.682	0.020	34.881	< 0.001 ***	1.977	1.903	2.054
Immunedeficiency	0.847	0.145	5.841	< 0.001 ***	2.331	1.755	3.097
Dementia	0.595	0.021	28.802	< 0.001 ***	1.812	1.740	1.887

* *p* < 0.05; *** *p* < 0.001

### 3.2 Breakdown of severe COVID-19 indices by exposure group

To further contextualize the findings, we examined the breakdown of individual severe outcomes (i.e., hospitalization, respiratory support, ECMO, and death) by exposure group (see **Table 3**). Among unexposed individuals 1.2% required hospitalization, 0.1% required respiratory support, <0.01% required ECMO, and 0.4% died. In the Valproate group rates were generally higher with hospitalization required in 3.8%, respiratory support in 0.3%, and death in 0.7%; ECMO use was not observed in this group. Among those exposed to lithium, hospitalization occurred in 0.6%, respiratory support in 0.1%, and death in 0.1%, with no ECMO cases recorded.

**Table 3:** Severity indices by exposure group.

Exposure	N	Hospitalization	Respiratory support	ECMO	Death
None, n (%)	1,484,479	17,610 (1.19)	1,474 (0.1)	281 (0.02)	5,720 (0.39)
Lithium, n (%)	857	5 (0.58)	1 (0.12)	0	1 (0.12)
Valproate, n (%)	5,302	201 (3.79)	17 (0.32)	0	38 (0.72)

Proportion of each severe outcome among those exposed vs. unexposed.

## 4 Discussion

In this large population-based study, lithium use was associated with significantly lower odds of severe COVID-19 compared with both non-exposed individuals and valproate users. This result is particularly notable given the older age and higher comorbidity burden in the lithium group. In contrast, valproate use was linked to increased odds of severe outcomes, though whether this reflects a direct harmful effect or simply the absence of lithium’s protective properties in an already vulnerable patient group remains an open question.

### 4.1 Interpretation and strengths

Several features of this study increase confidence in the findings. First, it is based on a large, representative, and real-world dataset from a single integrated healthcare system that covers over half of Israel’s population. The cohort included more than 1.4 million individuals with confirmed COVID-19, of whom nearly 6,200 were exposed to lithium or valproate. Importantly, lithium-treated patients were older and carried more comorbidities than the unexposed population, yet they experienced better outcomes. This counterintuitive pattern supports the interpretation of a genuine protective effect of lithium rather than a product of selection bias.

Second, the analytic strategy combined inverse probability weighting with Firth-penalized regression, addressing two central challenges: confounding due to baseline imbalances and quasi-separation from the low number of severe events in the lithium group. The consistency of results across both the composite outcome and the individual components (hospitalization, respiratory support, ECMO, death) further reinforces their robustness.

Third, exposure was defined stringently as continuous medication purchase throughout nearly the entire study period, distinguishing long-term treatment from transient use. This strengthens the inference that observed associations reflect sustained pharmacological influence rather than short-term prescribing.

Fourth, the inclusion of both valproate and non-exposed individuals as comparators is a notable strength. This design allowed us to separate lithium-specific effects from those related to mood stabilizer use in general or to psychiatric morbidity itself.

Fifth, the study period captures an era of high vaccine uptake (vaccinations began in Israel in December 2020 (18)), making the findings directly relevant to modern clinical practice. Observing a protective signal for lithium even in this context suggests that any benefit is not simply a relic of pre-vaccination viral dynamics, but may persist in the era of widespread immunization.

Finally, by examining severity rather than incidence, our study focuses on prognosis, which is of greater clinical importance, whereas prior work has mostly emphasized infection risk.

The results for valproate warrant a more cautious interpretation. On the surface, the doubling of risk among valproate users suggests harm. However, valproate users also tended to be older and sicker, and, unlike in the lithium group, there was no offsetting protective effect to counterbalance this vulnerability. In other words, the observed association with worse outcomes may reflect a combination of inherent clinical fragility and the absence of lithium’s immunomodulatory action, rather than a toxic effect of valproate per se. Distinguishing between these possibilities will require further mechanistic and prospective study.

### 4.2 Relation to prior evidence

Evidence regarding lithium and COVID-19 has been mixed. Small studies suggested possible benefit, while others raised concerns about toxicity. The largest data so far comes from two register studies. De Picker et al (15) reported a lower incidence of COVID-19 among lithium users compared with valproate users, consistent with our finding of a lithium-specific effect. More recently, Nilsson et al. conducted a Swedish nationwide register study of over 39,000 patients with bipolar disorder. In adjusted analyses, lithium was not associated with reduced mortality, hospitalization, or ICU admission (16). However, lithium monotherapy was linked to fewer hospitalizations and ICU admissions compared with combination therapy. Importantly, exposure was defined as collecting ≥2 lithium prescriptions in the preceding year. Given Swedish prescribing practices where stable patients often collect medication for several months at a time this definition likely captured most individuals on sustained treatment. Our study applied an even stricter exposure definition (≥5 of 6 quarterly purchases), reflecting Israeli prescribing requirements, where patients typically need a renewed prescription every 1–3 months. This operational difference may partly explain our stronger signal. The clearer signal in the Swedish monotherapy subgroup may reflect greater adherence to the treatment. In contrast, our study required near-continuous purchases throughout the observation period, a stricter definition that increases confidence that patients were actually on lithium when they contracted COVID-19. Additionally, by focusing specifically on the severity of outcomes rather than just infection risk, our study provides new, large-scale evidence that lithium may not only lower the likelihood of contracting an infection but also protect against a poor prognosis once an infection occurs. For valproate, our findings align with prior reports of increased risk and suggest that, at a minimum, it does not provide the immunological benefit associated with lithium.

### 4.3 Limitations

Several limitations should be acknowledged. First, as in all observational studies, residual confounding cannot be excluded. Although we adjusted for a wide range of demographic, medical, and psychiatric variables, unmeasured factors such as illness duration, psychiatric severity, or clinician prescribing preferences may have influenced both treatment choice and COVID-19 outcomes.

Second, exposure was defined by prescription purchase. While our definition of near-continuous purchasing provides greater confidence than less stringent approaches, it still cannot fully confirm adherence or therapeutic serum levels. Misclassification is therefore possible, although likely nondifferential and biasing results toward the null.

Third, the lithium group was relatively small compared with the unexposed cohort, and severe COVID-19 outcomes were rare. While we used Firth-penalized regression to handle quasi-separation, precision remains limited, and replication in other large datasets is needed.

Fourth, the study period ended in late 2021, before the Omicron variants became dominant and before widespread booster vaccinations were administered. As a result, generalizability to later phases of the pandemic may be limited.

Fifth, differences in testing behavior before diagnosis may also influence case inclusion. Individuals receiving lithium or valproate could have different thresholds for seeking testing when experiencing mild symptoms, which might affect the composition of the cohort. This factor cannot be evaluated directly in our dataset and represents a potential source of bias.

Sixth, psychotropic polypharmacy was not modeled. Lithium- and valproate-treated patients may differ in co-prescribed medications such as antipsychotics, antidepressants, or benzodiazepines, some of which have been associated with COVID-19 outcomes (8). Unequal distribution of these medications could contribute to observed group differences, particularly the less favorable outcomes in the valproate group. Modeling polypharmacy was beyond the scope of the present analysis but represents an important consideration for future work.

Finally, the analysis cannot determine causality. Our findings support an association between lithium use and reduced severity of COVID-19, but only randomized or mechanistic studies can confirm whether the effect is causal and identify underlying biological pathways.

### 4.4 Clinical and research implications

Clinically, these findings are reassuring for psychiatrists and patients alike. Lithium use was not associated with worse outcomes and, in fact, appeared protective, even in a group that was older and carried more medical comorbidities. For clinicians treating patients with severe mental illness, this provides support for the continued use of lithium during infectious outbreaks and reduces concern that it may confer additional risk in the context of COVID-19. By contrast, the elevated risks observed in valproate users highlight the need for vigilance, though our results suggest these may reflect underlying vulnerability rather than drug-specific harm.

Future research should aim to disentangle these possibilities. Randomized trials or pragmatic designs comparing lithium and valproate head-to-head could help clarify causality. Mechanistic studies examining immune function, viral replication, and inflammatory pathways in lithium- versus valproate-treated patients would be especially informative. Given lithium’s known neuroprotective properties, its potential role in mitigating long-COVID also deserves exploration.

In conclusion, lithium use was linked to substantially lower odds of severe COVID-19, even though lithium patients were older and carried more comorbidities than the unexposed population. Valproate users were likewise older and medically more fragile, and their worse outcomes may reflect this vulnerability combined with the absence of lithium’s protective effect, rather than clear evidence of valproate-related harm. Overall, these results highlight lithium’s potential role in reducing the severity of COVID-19 and suggest that the impact of psychotropic medications should be considered in relation to both psychiatric stability and broader physical health.

## Data Availability

The data analyzed in this study is subject to the following licenses/restrictions: The raw data supporting the conclusions of this article will be made available by the authors, without undue reservation. Requests to access these datasets should be directed to chen.avni@clalit.org.il.

## Footnotes

1 The sample included 10,801 patients diagnosed with bipolar disorder (see **Table 1**). The presence of a bipolar disorder diagnosis was not significantly associated with COVID-19 severity, this variable was excluded from the final regression model (see the Results).
